# Bioactive Compounds in Fermented Sausages Prepared from Beef and Fallow Deer Meat with Acid Whey Addition

**DOI:** 10.3390/molecules25102429

**Published:** 2020-05-22

**Authors:** Anna D. Kononiuk, Małgorzata Karwowska

**Affiliations:** Department of Meat Technology and Food Quality, University of Life Sciences in Lublin, 20-704 Lublin, Poland; anna.kononiuk@up.lublin.pl

**Keywords:** L-carnitine, conjugated linoleic acid, glutathione, peptides, beef, fallow deer meat, dry fermented sausage, acid whey

## Abstract

The present study examined the effect of the type of meat (beef and fallow deer) and the addition of freeze-dried acid whey on nutritional values and the content of bioactive compounds (peptides, L-carnitine, glutathione, and conjugated linoleic acid (CLA)) in uncured fermented sausages. The antioxidant properties of isolated peptides (ABTS, DPPH radical scavenging activity, and ferric-reducing antioxidant power) were also evaluated. The results showed that fallow deer sausages had higher peptide content than beef products. The addition of acid whey caused a decrease in the content of peptides, especially in fallow deer sausages. The glutathione content in beef sausages (22.91–25.28 mg 100 g^−1^ of sausage) was quite higher than that of fallow deer sausages (10.04–11.59 mg 100 g^−1^ of sausage). The obtained results showed a significantly higher content of CLA in beef sausages than in products from fallow deer meat. In conclusion, products prepared from fallow deer meat have generally higher nutritional value because of the content of peptides, their antioxidant properties, and the content of L-carnitine, while beef products have higher levels of CLA and glutathione.

## 1. Introduction

Growing awareness of food consumers about the relationship between diet, nutrients, and health has contributed to the development of new research focused on functional foods. Meat and meat products have a high nutritional value and are a good source of bioactive compounds (e.g., vitamins, minerals, peptides, and fatty acids) with a positive effect on human health [1]. Raw red meat is a rich source of many valuable, endogenous compounds such as high biological value proteins, essential amino acids, micronutrients, and bioactive compounds (carnitine, conjugated linoleic acid (CLA), endogenous antioxidants, and creatine) [2,3]. Available literature indicates their antioxidant and health-promoting properties, including immunomodulatory activity and protection against oxidative stress [4]. In recent years, game meat has received increasing interest from consumers. It is characterized by higher nutritional value than that of other meat, especially conventionally used beef or pork [5,6].

The functional value of meat can also be improved during processing. The use of fermentation and ripening to preserve meat avoids the loss of valuable compounds [7,8,9] as well as improve its functional value due to biochemical changes (generation of bioactive peptides and development of potentially probiotic bacterial strains) [10]. Lactic acid bacteria (LAB) are commonly used as a starter culture for the production of fermented sausages. Their application allows controlling the process and improving the quality and safety features of fermented sausages [11]. However, in traditional technology, starter cultures are rarely used, and fermentation is accomplished by natural flora. Previous studies also indicate the possibility of using acid whey as a natural source of LAB [12,13,14,15]. The addition of acid whey in dry fermented sausage production improves its properties and enhances its nutritional value by acting on the heme iron content and fatty acid composition. To the best of our knowledge, no studies have yet been published on the effect of whey on the content of bioactive compounds in fermented sausages.

The elimination of nitrate commonly used in meat processing as a curing agent also increases the nutritional value of meat products as its negative health effects are commonly described in the literature [16,17,18]. However, despite the positive health effects, the elimination of sodium nitrate has created some technological problems and may affect the quality characteristics of meat products.

Thus, the present study aimed to evaluate the effect of the type of meat (beef and fallow deer) on nutritional values and the content of bioactive compounds in organic uncured fermented sausages. The content of bioactive components of samples differing in additional substances was compared (with the traditional addition of sodium nitrite, without the addition of nitrate, and with different levels of addition of freeze-dried acid whey).

## 2. Materials and Methods

### 2.1. Raw Materials

Fallow deer meat and tallow were obtained from a certified organic breeding farm (Przytoczno Farm, Przytoczno, Poland), where animals live in natural environmental conditions and were slaughtered by a shot. Beef and beef fallow certified as organic were obtained from a butcher (Wasąg, Biłgoraj, Poland). As additives for sausage processing, sea salt, glucose, sodium nitrite, and freeze-dried acid whey were used. Sea salt (noniodinated and without anticaking agents; Cuore di mare, Italy) and glucose (Delecta, Poland) were purchased from local supermarkets (Lublin, Poland). Sodium nitrite (without anticaking agents) was obtained from StanLab (Lublin, Poland). Organic acid whey was bought fresh from a certified dairy product plant (R. Janowski, Ludwinów, Poland). It was obtained as a byproduct during the traditional production of cottage cheese. To limit variations in acid whey parameters, acid whey was first obtained, immediately frozen at −50 °C, and then freeze-dried (Labconco Free-Zone, Labconco Corporation, Kansas City, MO, USA). Acid whey powder was stored at −50 °C until sausage production. Shortly before use, an appropriate amount of acid whey powder was dissolved in saline for better distribution in stuffs. As described in our previous study [14], the pH of acid whey was 4.13 before and after freeze-drying. The LAB count was 4.58 ± 0.08 and 5.11 ± 0.06 cfu/mL before freezing and after freeze-drying, respectively.

### 2.2. Dry Fermented Sausage Preparation

Dry fermented sausages were prepared from raw materials obtained from fallow deer and beef. In the experiment, to produce one batch of sausage samples, 18 kg of beef and 2 kg of tallow, as well as 18 kg of fallow deer meat and 2 kg of tallow, were used. Meats and tallows were minced separately through a 10-mm grinding plate (Universal Machine KU2-3EK, Mesko-AGD, Skarżysko-Kamienna, Poland). Appropriate meat and tallow in the ratio of 9:1 were used for sausage preparation. Samples within the meat species were assigned to five batches according to Table 1.

Raw meat, fat, and additives were mixed and stuffed into fibrous casings (ø 65 mm, Viskase Corporation, Chicago, IL, USA). All variants were performed in two different batches as an experimental replication. Eight sausages of approximately 500 g were prepared for each sample for one batch. Sausages were weighted and hung in a temperature- and humidity-controlled chamber (16 °C, RH = 80–90%) until 30% weight loss (approximately 20 days). Samples were taken from each variant at the end of processing. Chemical composition; peptide content and its antioxidant activity; the content of amino acids, L-carnitine, and glutathione; and composition of fatty acids in the samples were determined.

### 2.3. Determination of Composition

The moisture, protein, and fat contents were determined according to PN ISO 1442:2000 [19], PN 75/A-04018 [20], and PN ISO 1444:2000 [21], respectively. Results were expressed as g per 100 g of product.

### 2.4. Determination of Peptides Content

Peptides were extracted according to the method of Zhu et al. [10] with slight modifications. Briefly, 5 g of sample was homogenized with 20 mL of 0.01 M HCl for 1 min with cooling ice by using a homogenizer (IKA T25, Staufen, Germany). The homogenate was centrifuged at 5000× *g* for 30 min at 4 °C, and after filtration through glass wool, 10 mL of supernatant was added to 30 mL of frozen ethanol. The mixture was kept at 4 °C overnight and then centrifuged at 5000× *g* for 30 min at 4 °C. The supernatant was collected and stored at −20 °C until it was concentrated in an evaporator. The concentrated extract was dissolved in 0.01 M HCl, filtered through a 0.45 μm nylon membrane filter (AlfaChem, Toruń, Poland), and stored at −20 °C prior to use. The content of peptide was measured by the *o*-phthaldialdehyde (OPA) spectrophotometric assay (Nicolet Evolution 300, Thermo Electron Corporation, Madison, WI, USA) [22]. Each sample was analyzed in triplicate. Leucine was used as a standard to quantify the peptide content. The content of peptide was calculated as mg of peptides per 100 g of product.

### 2.5. Peptides Antioxidant Activity

Peptide antioxidant activity was measured in terms of ABTS and DPPH radical scavenging activity and ferric-reducing antioxidant power (reducing power).

#### 2.5.1. ABTS^*+^ Radical Scavenging Activity

ABTS^*+^ radical scavenging activity was measured by the ABTS radical cation decolorization assay described by Re et al. [23]. The ability of the peptides to scavenge ABTS^*+^ radicals was evaluated by referring to the Trolox standard curve. The results were shown as the ability of peptides from 100 g of product to scavenge ABTS radical cations and expressed as equivalent mg Trolox per 100 g of product.

#### 2.5.2. DPPH Radical Scavenging Activity

DPPH radical scavenging activity of peptides isolated from sausages was assessed according to the method described by Zhu et al. [10]. The ability of peptides to scavenge DPPH free radicals was evaluated by referring to the Trolox standard curve. The results were shown as the ability of peptides from 100 g of product to scavenge DPPH free radicals and expressed as equivalent mg Trolox per mL.

#### 2.5.3. Ferric-Reducing Antioxidant Power (RP)

The ability of peptides to reduce iron from the Fe^3+^ (ferric) oxidation state to the Fe^2+^ (ferrous) oxidation state was determined by the method described by Mora et al. [24]. The results were calculated by referring to the results obtained for the ascorbic acid standard curve. The results were shown as the ability of peptides from 100 g of product to reduce iron from the Fe^3+^ to the Fe^2+^ oxidation state and expressed as equivalent mg ascorbic acid per mL.

### 2.6. Free Amino Acid Content

The contents of free amino acids were determined according to the method described by Stadnik and Dolatowski [25]. The following amino acids were monitored: phosphoserine, taurine, phosphoethanolamine, urea, aspartic acid, 4-hydroxy-proline, threonine, serine, asparagine, glutamic acid, 2-aminoadipic acid, proline, glycine, alanine, citrulline, 2-aminobutyric acid, valine, cystine, methionine, cystathionine, isoleucine, leucine, tyrosine, phenylalanine, β-alanine, 2-aminoisobutyric acid, γ−aminobutyric acid, ethanolamine, ornithine, lysine, histidine, 1-methylhistidine (1-MHis), 3-methylhistidine, and arginine. The results were expressed as mg per 100 g of product.

### 2.7. Determination of L-Carnitine Content

The content of L-carnitine was determined using the L-carnitine Assay Kit MAK063 (Sigma-Aldrich, St. Louis, MO, USA) according to a technical bulletin. The results were shown as mg of L-carnitine per 100 g of product.

### 2.8. Determination of Glutathione Content

Glutathione content was determined according to the assay described by Rahman et al. [26]. The results were expressed as mg of glutathione per 100 g of product.

### 2.9. Composition of Fatty Acids

Fatty acid profile was determined by gas chromatography after conversion of the fats to fatty acid methyl esters (FAME) [27]. The method of Folch et al. [28] was used to extract lipids from the sausage samples. A gas chromatographic analysis was performed using a chromatograph (Varian 450-GC, Walnut Creek, CA, USA) equipped with a capillary column (Select Biodiesel for FAME, Varian, Palo Alto, CA, USA, 30 m × 0.32 mm × 0.25 μm film thickness). Injector and detector temperatures were 250 °C and 300 °C, respectively. After injection, the column temperature was programmed to increase to 200 °C for 10 min, subsequently increased to 240 °C at the rate of 3 °C min^−1^, and then held at the final temperature for 4 min. Helium was used as a carrier gas (3 mL min^−1^). The amounts of fatty acids were calculated from the chromatograms and from an internal standard containing FAME.

### 2.10. Statistical Analysis

The collected data were analyzed using Statistica version 13.3 software (Dell Inc. Round Rock, TX, USA) and are expressed as mean ± standard error. The experiment was conducted in two independent batches, included in the model as a random term. Each batch included 40 sausages (8 pieces for each sample). All measurements were performed in three repeats for each piece of samples. The differences between the batches were not significant. Effects between categorical factors (day, type of meat, and variant) and variables between the subgroups were analyzed by a factorial ANOVA. Homogeneity of variances was assessed by Levene’s test. Post hoc comparisons were performed using Tukey’s test. All differences were significant at *p* ≤ 0.05. Pearson’s correlation coefficient was calculated to determine the strength of the relationship between variables.

## 3. Results

### 3.1. Sausages Composition

Analysis of sausage composition (Table 2) showed that beef sausages had a higher content of protein (from 36.11 to 38.91 g 100 g^−1^ of product) and fat (from 23.36 to 27.96 g 100 g^−1^ of product) than fallow deer sausages (30.30–31.05 and 12.79–15.09 g 100 g^−1^ of product, respectively). All variants of fallow deer meat sausages had approximately 10–15% less fat than the corresponding beef sausage variants. Differences in protein and intramuscular fat content between beef and fallow deer sausages were due to the different sources of raw meat [5]. There were no significant effects of additives used on changes in sausage composition. Although the same conditions were applied during the fermentation process, sausages prepared from fallow deer meat had lower weight loss (higher moisture content).

### 3.2. Peptide Content and Their Antioxidant Activity

Table 3 shows the results of peptide content and antioxidant activity of peptides obtained from beef and fallow deer fermented sausages. The results revealed that fallow deer sausages had a higher content of peptide than beef sausages. The addition of acid whey significantly influenced the peptide content of fallow deer sausages. Samples with acid whey addition (SAW, SAW2, and SAW4) had a significantly lower content of peptide (1896.30, 1759.41, and 1687.79 mg 100 g^−1^ of product, respectively) than reference (S, 2004.88 mg 100 g^−1^ of product) or control sample (C, 2061.48 mg 100 g^−1^ of product). The results indicated a high level of the proteolysis process due to the presence of LAB and lactic acid in acid whey. Despite the significantly lower content of peptides in beef sausages (from 1512.25 to 1695.03 mg 100 g^−1^ of product), the DPPH antioxidant activity was higher in these sausages. Peptides from 100 g of beef sausages showed antioxidant activity between 4.78 and 7.51 mg Trolox, whereas in sausages from fallow deer, the DPPH radical scavenging activity was between 2.72 and 3.88 mg Trolox.

Table 4 shows correlations between peptide content (influenced by different types of meat used and variants) and antioxidant activity (measured by different assays). The results show that the antioxidant activity of peptides against ABTS and DPPH were highly positively related to the peptide content of the different variants of sausages, whereas RP was negatively related to the peptide content. Moreover, changes in the peptide content based on the type of meat did not correlate with RP changes and with changes in ABTS radical scavenging activity.

### 3.3. L-Carnitine and Glutathione Content

According to the results shown in Table 5, the levels of L-carnitine in fallow deer sausages were almost twice as high as those in beef sausages. Additionally, fallow deer sausages were also richer sources of lysine and methionine (Table 6), which are the precursors for the endogenous synthesis of L-carnitine. The use of freeze-dried acid whey did not significantly affect the level of L-carnitine in the different variants of both sausages. Similarly, there was no observed effect of the additive used on glutathione content. The glutathione content in beef sausages (22.91–25.28 mg 100 g^−1^ of sausage) was more than twice as high as that in fallow deer sausages (10.04–11.59 mg 100 g^−1^ of sausage).

### 3.4. Free Amino Acid Profile

Table 6 shows the amino acid profile of the studied sausages. The total content of amino acids was lower in beef sausages with acid whey addition (SAW, 314.24 ± 5.70 mg 100 g^−1^ of product) but was higher in fallow deer reference sample (with sea salt addition, S, 920.89 ± 13.65 mg 100 g^−1^ of product). Nevertheless, in both groups (beef and fallow deer meat sausages), the reference sample (S, with sea salt addition) was the variant with the highest amount of free amino acids. Among the free amino acids evaluated, phosphoserine, phosphoethanolamine, urea, 4-hydroxy-proline, 2-aminoadipic acid, proline, citrulline, 2-aminobutyric acid, cystathionine, β-alanine, 2-aminoisobutyric acid, and 3-methylhistidine were not identified in any of the test samples. In the tested samples, the most abundant amino acid was leucine, with content ranging from 33.06 ± 0.23 (SAW) to 62.27 ± 2.21 (S) for beef samples and from 72.85 ± 1.63 (SAW2) to 121.18 ± 1.54 (S) for fallow deer meat samples.

The content of taurine (which is considered as a bioactive compound) was significantly higher in the fallow deer meat samples (except variant SAW2: with a double portion of acid whey). With a few exceptions, fallow deer sausages were a rich source of most free amino acids such as aspartic acid, serine, glycine, alanine, valine, cystine, methionine, isoleucine, leucine, phenylalanine, γ-aminobutyric acid, ornithine, lysine, and arginine. Arginine was detected only in fallow deer sausages. Higher content of 1-MHis was detected in beef sausages (49.43–95.11 mg 100 g^−1^ of product) than in fallow deer sausages (35.65–53.94 mg 100 g^−1^ of product). This indicated a higher level of anserine in this type of meat because high levels of 1-MHis tend to inhibit the enzyme carnosinase (which splits anserine into β-alanine and 1-MHis) and increase anserine levels [29].

### 3.5. Fatty Acid Profile and Nutritive Characteristic of Tested Sausages

The analysis of the main fractions of fatty acids (Table 7) did not indicate any effect of the additives used. However, the type of meat used significantly affected the intake of different fatty acids. Fallow deer sausages showed approximately 20% higher content of saturated fatty acids, which was recompensed by lower content (also approximately 20%) of monounsaturated fatty acids in comparison to beef sausages. Polyunsaturated fatty acids content was significantly higher in fallow deer sausages than in beef sausages.

Although fatty acids profile (Table 8) was similar for all the variants of both types of meats, small differences in acids C8:0 and C10:0 were observed. In all cases, the fatty acids content increased in the following order: C (control sample) < S (reference sample) < SAW (sample with one portion of acid whey) < SAW2 (sample with a double portion of acid whey) < SAW4 (sample with a quadruple portion of acid whey). Differences in fatty acids profile between beef sausages and fallow deer sausages were more evident. Beef sausages had a clearly lower contribution of lauric (C12:0), linoleic (C18:2), arachidic (C20:0), behenic (C22:0), arachidonic (C20:4), and docosahexaenoic (DHA, C22:6) acids and higher contribution of palmitoleic (C16:1), elaidic (C18:1), and linolenic (C18:3) acids in comparison to fallow deer sausages.

Table 8 shows the proportion of different fractions of fatty acids. Both beef and fallow deer meat sausages contained a higher proportion of SFA than PUFA. However, beef sausages had a higher contribution of UFA in the UFA/SFA ratio than that in fallow deer sausages. The analysis of the MUFA/SFA and PUFA/SFA ratios indicated that the difference was mainly observed for changes in MUFA content of the fatty acids (the PUFA/SFA ratio was similar for both types of sausages). Nevertheless, fatty acids of fallow deer sausages had the recommended n-6:n-3 ratio (4:1), whereas the ratio was approximately 2.5:1 in beef sausages.

Ruminants’ meat is an important source of CLA [30]. The main CLA isomer found in beef and fallow deer meat sausages was *cis*9-*trans*11, which is also called rumenic acid, followed by *cis*9-*cis*11, *trans*9-*trans*11, and *trans*10-*cis*12 (Table 9). The concentration of CLA *cis*9-*trans*11 isomers in sausage samples ranged from 0.100% to 0.237%. The obtained results showed a significantly higher content of CLA in beef sausages than in fallow deer meat sausages. Furthermore, in fallow deer sausages, samples with acid whey (SAW, SAW2, and SAW4) had a significantly higher content of most CLA isomers than other experimental samples.

## 4. Discussion

The present research assessed the impact of the type of meat and freeze-dried acid whey addition on the quality of fermented sausages. Fermentation of meat products is an ancestral tradition passed onto new generations. The main objective of this cooking method is to extend the shelf life of the meat product. The results of selected parameters related to food safety of fallow deer and beef uncured fermented sausages with freeze-dried acid whey addition have already been published [14]. It has been shown that fermentation decreases the pH of the products, especially for sausages made of fallow deer meat. After fermentation, the products show low water activity (between 0.88 and 0.920). Physicochemical changes during production affected the chemical composition of the products presented in this paper. The significant loss of water during production implied that the sausages had a high protein content. According to Regulation (EC) No. 1924/2006 [31], both types of sausages meet the requirements for high-protein products (31–42% and 48–52% of the energy value is provided by protein in beef and fallow deer sausages, respectively). Moreover, previously published results [14] showed an increase in the content of LAB after the production process, which indicated the occurrence of fermentation.

The fermentation process also has many benefits as it improves the functional value of meat due to biochemical changes. Meat fermentation leads to the splitting of protein into peptides and amino acids. Statistical analysis performed in this study indicated a positive correlation between peptides and the content of amino acids formed due to proteolysis, one of the main mechanisms that occur during the ripening of dry fermented meat products. The peptides originating from meat show various biological activities that are important for human health. Many studies have described the generation of peptides with antioxidant and antihypertensive activities during the processing of different types of sausages [32,33,34]. The antioxidant activity of peptides in meat products is very important because of their natural potential to influence human health as well as their positive effect on food quality, such as inhibition of oxidative reactions, which increases the shelf life of products [35]. The results of our study show that fallow deer sausages had a higher content of peptides than beef sausages. This may be due to the higher content of protein in venison [5] as well as intrinsic factors that influence the proteolysis activity in each type of meat. Despite significantly lower content of peptides in beef sausages, the DPPH antioxidant activity was higher in these sausages. Peptides from beef sausages showed antioxidant activity (measured as DPPH radical scavenging activity) almost two times higher than that in sausages from fallow deer. According to Zheng et al. [36], the difference is related to the presence of free cysteine and peptides containing it. The lipid-soluble radical DPPH^•^ is very sensitive for substances containing strong reducing functional groups in their molecules, such as the free -SH in Cys [36]; this indicated that peptides obtained from beef sausages contain a higher amount of cysteine than those obtained from fallow deer sausages.

L-carnitine plays a key role in metabolism. It is essential for the transport of activated long-chain fatty acids from the cytosol to the mitochondria to make it available for β-oxidation. Additionally, L-carnitine participates in the oxidation of fatty acids in peroxysomes, maintenance of CoA/acyl-CoA ratio in the cell, and utilization of ketone body. Thus, L-carnitine deficiency may lead to disorders of metabolic processes affecting muscle and heart function and manifests as myopathy or heart disease [37]. In the human body, carnitine is endogenously synthesized from the amino acids lysine and methionine (which may come from dietary sources as well as from endogenous protein degradation). Nevertheless, endogenous synthesis of L-carnitine may not be adequate in people with a stressful or physically active lifestyle as well as in individuals with genetic disorders or some chronic diseases linked to the disrupted synthesis of L-carnitine [37,38]. The richest sources of carnitine in the human diet are meat (especially red meat such as beef) and dairy products (milk and curd cheese) [39]. In processed meat, the carnitine level is much lower (2.9–66.3 mg 100 g^−1^ of Andouillette (sausage of offal) and merquez (beef sausage)) [40]. Bioavailability of dietary L-carnitine depends on the amount of L-carnitine in the meal and ranges from 54% to 87%, while the bioavailability of L-carnitine in dietary supplements is 14–18% [41]. According to the results of the current study, the levels of L-carnitine in fallow deer sausages were almost twice as high as those in beef sausages. Additionally, fallow deer sausages are also richer sources of lysine and methionine, which are the precursors for the endogenous synthesis of L-carnitine. Previous studies have confirmed that the contents of methionine, lysine, and L-carnitine are positively correlated. The use of freeze-dried acid whey did not significantly affect the level of L-carnitine in the different variants of both sausages. Knüttel-Gustavsen and Harmeyer [39] showed that the content of L-carnitine in beef sausages (70.66–83.27 mg 100 g^−1^ of product) was slightly higher than that in raw beef steak (661 mg kg^−1^ wet weight) due to the difference in water content (content of L-carnitine in dry matter of beef steak was 2320 mg kg^−1^ of dry matter). Despite these differences in water content, the L-carnitine level of fallow deer sausages (135.70–152.02 mg 100 g^−1^ of product) is even higher than the L-carnitine content in dry matter of beef steak, which confirms that fallow deer meat is a valuable source of this compound [39].

Another bioactive compound whose main source is meat is glutathione (tripeptide of cysteine, glycine, and glutamic acid) that plays a key role in the reduction of oxidative stress by maintaining reduction-oxidation homeostasis, metabolic detoxification, and regulation of the immune system. Deficiency or reduction of glutathione content may be related to the occurrence of chronic diseases [42] such as neurodegeneration [43], hypertension [44], liver diseases, and diabetes [45]. Literature sources [4] report that the glutathione level in our body decreases with age due to the decreasing glutamyl cysteine synthetase activity; thus, its level may be supplemented by diet. Research indicates that daily oral intake of 250–1000 mg of glutathione leads to an increase in body stores, decreases the markers of oxidative stress, and increases the cytotoxicity level of natural killer cells [46]. It is difficult to find glutathione content in food products investigated in recent studies. However, Bukowska et al. [47] in their review indicated that the richer sources of glutathione are asparagus, cooked ham, and fried beef, which contain 28.3, 23.3, and 17.3 mg of glutathione 100 g^−1^ of product, respectively. Our research indicated that beef is a richer source of glutathione than fallow deer meat. The glutathione content in beef sausages was more than twice as high as that in fallow deer sausages. Acid whey or sodium nitrite addition showed no effect on glutathione content.

The fatty acid profile in meat and meat products is very important because it determines their nutritional value. PUFA consumption is recommended to constitute 5–10% energy from n-6 and 0.6–1.2% energy from n-3, with not less than 0.5% energy from α-linolenic acid (ALA) and 250 mg per day of eicosapentaenoic acid (EPA) and docosahexaenoic acid (DHA) [48]. An excess of n-6 PUFA can interfere with the metabolism of n-3 PUFA and reduce their incorporation into tissue lipids. This represents a risk factor for many human pathologies [49]. Thus, the PUFA/SFA and n-6/n-3 PUFA ratios have become some of the most significant parameters in evaluating the nutritional value and healthfulness of foods [50]. The results of the present study showed that both beef and fallow deer meat sausages contain a higher proportion of SFA than PUFA, which is consistent with the finding of Muguerza et al. [51]. The PUFA/SFA ratios obtained in this study were similar for both types of sausages. A very important factor that determines the nutritional value of animal fat is the content of CLA. CLA refers to a group of PUFA that exist as positional and stereo-isomers of conjugated dienoic octadecadienoate. The most commonly recommended daily intake of CLA for adults is 0.8 g per day [52]. In the current study, we hypothesized that LAB present in acid whey can produce CLA isomers from linoleic acid. This hypothesis has not been confirmed for beef sausages similar to that in a previous study [53]. However, in fallow deer meat sausages, samples with acid whey had a significantly higher content of CLA cis9-cis11 and CLA cis 9-trans11 isomers than other experimental samples.

## 5. Conclusions

Sausages made from beef and fallow deer meat are the source of bioactive compounds, including peptides, L-carnitine, glutathione, and CLA isomers. However, the results of our experiment show that products prepared from fallow deer meat have a higher nutritional value in terms of peptide content and their antioxidant properties and L-carnitine content. Moreover, the addition of acid whey improves the CLA content of dry fermented sausages prepared from fallow deer meat, thereby improving their nutritional value.

## Figures and Tables

**Table 1:** Additives used for each variant of sausage.

Sample	Glucose (% *w*/*w*)	Sea Salt (% *w*/*w*)	Sodium Nitrite (% *w*/*w*)	Acid Whey Powder (% *w*/*w*)	Water/Saline (% *w*/*w*)
C	0.6	2.786	0.014		5
S	0.6	2.8			5
SAW	0.6	2.8		0.35	5
SAW2	0.6	2.8		0.70	5
SAW4	0.6	2.8		1.40	5

C—sample with addition of sea salt and sodium nitrite; S—sample with addition of sea salt; SAW—sample with addition of sea salt and 0.35% *w*/*w* freeze-dried acid whey; SAW2—sample with addition of sea salt and 0.70% *w*/*w* freeze-dried acid whey; SAW4—sample with addition of sea salt and 1.40% *w*/*w* freeze-dried acid whey.

**Table 2:** Composition of fermented sausages (% *w*/*w*) made from fallow deer and beef meat with different additives (mean ± standard error).

Parameter	Variant	Type of Meat		M	V	M × V
		Beef	Fallow Deer			
Protein (g 100 g^−1^ of product)				***	***	***
	C	38.91 ^a^ ± 0.28	30.30 ^c^ ± 0.13			
	S	36.11 ^b^ ± 0.15	31.05 ^c^ ± 0.17			
	SAW	38.06 ^a^ ± 0.25	30.47 ^c^ ± 0.17			
	SAW2	38.67 ^a^ ± 0.21	30.49 ^c^ ± 0.21			
	SAW4	36.48 ^b^ ± 0.16	30.54 ^c^ ± 0.19			
Fat (g 100 g^−1^ of product)				***	***	***
	C	23.36 ^c^ ± 0.14	13.59 ^e^ ± 0.58			
	S	27.96 ^a^ ± 0.13	12.79 ^e^ ± 0.10			
	SAW	25.78 ^b^ ± 0.18	13.87 ^de^ ± 0.52			
	SAW2	27.66 ^a^ ± 0.12	15.09 ^d^ ± 0.18			
	SAW4	27.09 ^ab^ ± 0.15	13.77 ^de^ ± 0.24			
Moisture (g 100 g^−1^ of product)				***	***	***
	C	37.21 ^c^ ± 0.19	54.35 ^a^ ± 0.32			
	S	35.83 ^d^ ± 0.16	53.72 ^ab^ ± 0.31			
	SAW	36.32 ^cd^ ± 0.17	54.45 ^a^ ± 0.44			
	SAW2	34.02 ^e^ ± 0.17	52.95 ^b^ ± 0.42			
	SAW4	36.24 ^cd^ ± 0.18	52.64 ^b^ ± 0.26			

C—sample with addition of sea salt and sodium nitrite; S—sample with addition of sea salt; SAW—sample with addition of sea salt and 0.35% *w*/*w* freeze-dried acid whey; SAW2—sample with addition of sea salt and 0.70% *w*/*w* freeze-dried acid whey; SAW4—sample with addition of sea salt and 1.40% *w*/*w* freeze-dried acid whey. ^a–e^ Means within one variable followed by the same letters did not differ significantly (*p* ≥ 0.05). Significance of fixed effects, M—type of meat, V—variant, M × V—interaction between them, *p*-value: *** (*p* < 0.001), ** (*p* < 0.01), * (*p* < 0.05), n.s. (not significant).

**Table 3:** Content of peptide in fermented sausages made from beef and fallow deer meat with different additives and their antioxidant activity measured as ability to scavenge DPPH and ABTS^+^ radical and reducing power (RP, mean ± standard error).

Parameter	Variant	Type of Meat		M	V	M × V
		Beef	Fallow Deer			
Peptide content (g 100 g^−1^ of product)				***	***	***
	C	1602.41 ^def^ ± 25.79	2061.48 ^a^ ± 14.71			
	S	1695.03 ^cd^ ± 18.28	2004.88 ^a^ ± 32.21			
	SAW	1786.51 ^c^ ± 33.20	1896.30 ^b^ ± 21.78			
	SAW2	1589.15 ^ef^ ± 9.07	1759.41 ^c^ ± 13.85			
	SAW4	1512.25 ^f^ ± 18.46	1687.79 ^cde^ ± 14.25			
ABTS (mg Trolox eqv. 100 g^−1^ of product)				***	***	***
	C	6.98 ^cde^ ± 0.24	7.22 ^cd^ ± 0.22			
	S	6.16 ^e^ ± 0.11	9.73 ^a^ ± 0.10			
	SAW	7.45 ^c^ ± 0.07	9.16 ^ab^ ± 0.19			
	SAW2	7.23 ^cd^ ± 0.24	8.48 ^b^ ± 0.10			
	SAW4	6.43 ^de^ ± 0.20	9.15 ^ab^ ± 0.22			
DPPH (mg Trolox eqv. 100 g^−1^ of product)				***	***	***
	C	7.51 ^a^ ± 0.27	2.72 ^f^ ± 0.09			
	S	4.78 ^cd^ ± 0.16	2.74 ^f^ ± 0.18			
	SAW	6.26 ^b^ ± 0.11	3.88 ^de^ ± 0.19			
	SAW2	5.37 ^bc^ ± 0.51	3.20 ^ef^ ± 0.08			
	SAW4	5.79 ^bc^ ± 0.07	3.69 ^ef^ ± 0.13			
RP (mg ascorbic acid eqv. 100 g^−1^ of product)				***	***	n.s.
	C	27.18 ^e^ ± 0.49	43.00 ^ab^ ± 0.55			
	S	29.43 ^d^ ± 0.20	43.35 ^ab^ ± 0.64			
	SAW	31.75 ^c^ ± 0.29	44.80 ^a^ ± 0.23			
	SAW2	27.85 ^de^ ± 0.28	41.88 ^b^ ± 0.52			
	SAW4	28.53 ^de^ ± 0.20	42.92 ^b^ ± 0.23			

C—sample with addition of sea salt and sodium nitrite; S—sample with addition of sea salt; SAW—sample with addition of sea salt and 0.35% *w*/*w* freeze-dried acid whey; SAW2—sample with addition of sea salt and 0.70% *w*/*w* freeze-dried acid whey; SAW4—sample with addition of sea salt and 1.40% *w*/*w* freeze-dried acid whey. ^a–f^ Means within one variable followed by the same letters did not differ significantly (*p* ≥ 0.05). Significance of fixed effects, M—type of meat, V—variant, M × V—interaction between them, *p*-value: *** (*p* < 0.001), ** (*p* < 0.01), * (*p* < 0.05), n.s. (not significant).

**Table 4:** Pearson’s correlation coefficient of peptides antioxidant activity measured by three assays (ABTS, DPPH, and RP) and peptide content from fallow deer (FD) and beef (B) sausages made with different additives.

Antioxidant Activity Assay	Peptide Content						
	Type of Meat		Variant				
	B	FD	C	S	SAW	SAW2	SAW4
ABTS	**0.37**	−0.21	0.40	**0.94**	**0.74**	**0.89**	**0.90**
DPPH	**0.76**	**−0.58**	**0.98**	**0.96**	**0.67**	**0.97**	**0.94**
RP	0.03	0.33	**−0.93**	**−0.95**	**−0.64**	**−0.74**	**−0.83**

Correlations in bold are significant at *p* < 0.05.

**Table 5:** Content of L-carnitine and glutathione in dry fermented sausages made from fallow deer and beef meat with different additives (mean ± standard error).

Parameter	Variant	Type of Meat		M	V	M × V
		Beef	Fallow Deer			
L-carnitine (mg 100 g^−1^ of product)				***	***	*
	C	70.66 ^e^ ± 1.08	135.70 ^c^ ± 1.77			
	S	81.87 ^d^ ± 1.15	140.98 ^bc^ ± 1.28			
	SAW	79.52 ^de^ ± 1.31	152.02 ^a^ ± 3.66			
	SAW2	83.27 ^d^ ± 1.35	140.93 ^bc^ ± 0.95			
	SAW4	76.49 ^de^ ± 3.13	145.94 ^ab^ ± 1.56			
Glutathione (mg 100 g^−1^ of product)				***	***	***
	C	25.28 ^a^ ± 0.39	10.54 ^e^ ± 0.06			
	S	22.91 ^c^ ± 0.22	11.59 ^d^ ± 0.05			
	SAW	23.76 ^b^ ± 0.13	11.51 ^d^ ± 0.06			
	SAW2	25.09 ^a^ ± 0.11	10.70 ^e^ ± 0.11			
	SAW4	22.96 ^c^ ± 0.10	10.04 ^e^ ± 0.10			

C—sample with addition of sea salt and sodium nitrite; S—sample with addition of sea salt; SAW—sample with addition of sea salt and 0.35% *w*/*w* freeze-dried acid whey; SAW2—sample with addition of sea salt and 0.70% *w*/*w* freeze-dried acid whey; SAW4—sample with addition of sea salt and 1.40% *w*/*w* freeze-dried acid whey. ^a–e^ Means within one variable followed by the same letters did not differ significantly (*p* ≥ 0.05). Significance of fixed effects, M—type of meat, V—variant, M × V—interaction between them, *p*-value: *** (*p* < 0.001), ** (*p* < 0.01), * (*p* < 0.05), n.s. (not significant).

**Table 6:** Content of free amino acids (mg 100 g^−1^ of product) in fermented sausages from beef and fallow deer meat with different addition of acid whey (mean ± standard error).

Meat	Beef					Fallow Deer				
Variant	C	S	SAW	SAW2	SAW4	C	S	SAW	SAW2	SAW4
Taurine	17.82 ^ef^ ± 0.39	25.47 ^bc^ ± 1.41	12.57 ^g^ ± 0.07	22.98 ^cd^ ± 0.44	16.31 ^f^ ± 0.94	24.43 ^bc^ ± 1.03	31.85 ^a^ ± 0.17	26.84 ^b^ ± 0.17	19.87 ^de^ ± 0.84	23.09 ^cd^ ± 0.10
Aspartic acid	12.02 ^e^ ± 0.95	10.47 ^ef^ ± 0.79	6.35 ^g^ ± 0.07	11.02 ^ef^ ± 0.08	9.02 ^fg^ ± 0.66	23.95 ^b^ ± 0.51	29.30 ^a^ ± 0.23	19.24 ^c^ ± 0.89	16.62 ^cd^ ± 0.61	15.32 ^d^ ± 0.12
Threonine	16.38 ^f^ ± 0.08	22.32 ^d^ ± 1.18	8.65 ^g^ ± 0.58	19.39 ^e^ ± 0.77	9.46 ^g^ ± 0.47	27.50 ^c^ ± 0.16	38.57 ^a^ ± 0.74	33.71 ^b^ ± 0.21	21.93 ^de^ ± 0.60	21.13 ^de^ ± 0.24
Serine	16.12 ^bc^ ± 0.18	7.00 ^de^ ± 0.12	2.51 ^e^ ± 0.40	7.02 ^de^ ± 0.02	1.33 ^e^ ± 0.11	21.77 ^b^ ± 2.76	32.30 ^a^ ± 4.19	18.28 ^bc^ ± 0.29	11.66 ^cd^ ± 0.50	13.17 ^cd^ ± 0.10
Glutamic acid	56.63 ^bc^ ± 1.01	76.07 ^a^ ± 4.11	40.07 ^cd^ ± 0.13	68.36 ^ab^ ± 0.22	56.00 ^bc^ ± 3.16	45.50 ^cd^ ± 5.66	64.03 ^ab^ ± 9.08	28.69 ^d^ ± 0.57	29.97 ^d^ ± 1.91	35.66 ^d^ ± 0.23
Glycine	15.33 ^ef^ ± 0.05	18.41 ^d^ ± 1.30	9.81 ^g^ ± 0.08	18.04 ^d^ ± 0.71	13.02 ^f^ ± 0.40	24.00 ^c^ ± 0.53	35.46 ^a^ ± 0.20	30.40 ^b^ ± 0.37	17.92 ^de^ ± 0.39	17.37 ^de^ ± 0.49
Alanine	52.22 ^e^ ± 1.02	73.97 ^cd^ ± 3.49	36.31 ^f^ ± 0.14	68.17 ^d^ ± 0.41	48.70 ^e^ ± 2.34	80.34 ^c^ ± 0.84	116.06 ^a^ ± 1.95	99.70 ^b^ ± 0.83	67.88 ^d^ ± 2.31	69.36 ^d^ ± 0.53
Valine	34.90 ^e^ ± 0.34	41.14 ^c^ ± 1.75	21.13 ^g^ ± 0.21	38.52 ^cde^ ± 0.40	27.68 ^f^ ± 1.44	40.47 ^cd^ ± 0.67	58.84 ^a^ ± 0.62	52.47 ^b^ ± 1.04	35.19 ^e^ ± 1.39	36.36 ^de^ ± 0.17
Cystine	n.d.	5.18 ^bc^ ± 0.17	3.10 ^d^ ± 0.18	4.28 ^c^ ± 0.40	5.50 ^ab^ ± 0.30	5.58 ^ab^ ± 0.17	5.74 ^ab^ ± 0.03	6.36 ^a^ ± 0.04	5.76 ^ab^ ± 0.11	4.52 ^c^ ± 0.24
Methionine	16.63 ^f^ ± 0.06	21.28 ^e^ ± 0.30	10.57 ^g^ ± 0.09	19.99 ^e^ ± 0.51	15.62 ^f^ ± 0.54	27.49 ^c^ ± 0.13	40.61 ^a^ ± 0.21	34.96 ^b^ ± 0.17	25.03 ^d^ ± 0.56	24.89 ^d^ ± 0.19
Isoleucine	22.19 ^e^ ± 0.11	27.23 ^d^ ± 0.81	15.02 ^f^ ± 0.18	27.25 ^d^ ± 0.63	20.02 ^e^ ± 0.90	30.95 ^c^ ± 0.20	48.32 ^a^ ± 0.99	43.42 ^b^ ± 0.14	27.73 ^d^ ± 0.88	28.55 ^cd^ ± 0.10
Leucine	51.33 ^f^ ± 0.49	62.27 ^e^ ± 2.21	33.06 ^h^ ± 0.23	59.21 ^e^ ± 1.24	44.24 ^g^ ± 2.26	82.84 ^c^ ± 0.28	121.18 ^a^ ± 1.54	108.58 ^b^ ± 0.38	72.85 ^d^ ± 1.63	74.92 ^d^ ± 0.51
Tyrosine	n.d.	n.d.	n.d.	n.d.	1.50 ± 0.14	n.d.	n.d.	n.d.	n.d.	n.d.
Phenylalanine	35.15 ^e^ ± 0.86	39.25 ^d^ ± 1.29	23.03 ^g^ ± 0.31	36.70 ^de^ ± 0.66	30.53 ^f^ ± 1.75	45.07 ^c^ ± 0.28	63.14 ^a^ ± 0.86	50.88 ^b^ ± 0.17	38.20 ^de^ ± 0.84	38.82 ^de^ ± 0.26
γ–aminobutyric acid	6.12 ^c^ ± 0.02	2.62 ^c^ ± 0.14	2.25 ^c^ ± 0.18	3.44 ^c^ ± 0.03	2.76 ^c^ ± 0.33	21.63 ^b^ ± 4.16	29.94 ^b^ ± 6.33	50.68 ^a^ ± 0.24	24.88 ^b^ ± 0.61	19.43 ^b^ ± 0.08
Ethanolamine	2.99 ^b^ ± 0.05	1.86 ^b^ ± 0.05	12.98 ^a^ ± 4.01	2.74 ^b^ ± 0.05	2.68 ^b^ ± 0.07	2.88 ^b^ ± 0.13	2.40 ^b^ ± 0.13	2.43 ^b^ ± 0.08	2.49 ^b^ ± 0.04	2.41 ^b^ ± 0.01
Ornithine	8.08 ^f^ ± 0.08	5.98 ^g^ ± 0.16	4.29 ^h^ ± 0.35	7.76 ^f^ ± 0.04	3.97 ^h^ ± 0.15	25.80 ^c^ ± 0.25	37.41 ^a^ ± 0.37	35.51 ^b^ ± 0.18	19.27 ^d^ ± 0.70	14.92 ^e^ ± 0.26
Lysine	23.03 ^f^ ± 0.21	24.04 ^f^ ± 0.24	15.67 ^g^ ± 0.74	28.53 ^e^ ± 1.02	27.73 ^e^ ± 0.95	46.34 ^c^ ± 1.03	68.45 ^a^ ± 0.57	54.15 ^b^ ± 0.88	38.20 ^d^ ± 0.98	44.07 ^c^ ± 0.27
Histidine	15.82 ^d^ ± 0.65	15.27 ^d^± 0.10	7.45 ^h^ ± 0.42	14.18 ^de^ ± 0.39	9.49 ^g^ ± 0.28	17.58 ^c^ ± 0.26	25.64 ^a^ ± 0.31	22.14 ^b^ ± 0.29	12.86 ^ef^ ± 0.57	12.42 ^f^ ± 0.04
1-methylhistidine	70.53 ^b^ ± 1.16	95.11 ^a^ ± 3.15	49.43 ^c^ ± 0.17	90.81 ^a^ ± 0.83	65.63 ^b^ ± 1.93	37.06 ^d^ ± 0.12	53.94 ^c^ ± 0.19	48.38 ^c^ ± 0.38	35.65 ^d^ ± 0.79	40.17 ^d^ ± 0.29
Arginine	n.d.	n.d.	n.d.	n.d.	n.d.	9.67 ^d^ ± 0.23	17.73 ^a^ ± 0.10	13.28 ^b^ ± 0.31	11.36 ^c^ ± 0.32	18.33 ^a^ ± 0.10
Total	473.10 ^e^ ± 7.32	574.94 ^d^ ± 22.64	314.24 ^g^ ± 5.70	548.39 ^d^ ±.4.42	411.19 ^f^ ± 18.76	640.83 ^c^ ± 7.15	920.89 ^a^ ± 13.65	780.11 ^b^ ± 2.92	535.33 ^d^ ± 16.56	554.90 ^d^ ± 2.34

n.d. (not detected). C—sample with addition of sea salt and sodium nitrite; S—sample with addition of sea salt; SAW—sample with addition of sea salt and 0.35% *w*/*w* freeze-dried acid whey; SAW2—sample with addition of sea salt and 0.70% *w*/*w* freeze-dried acid whey; SAW4—sample with addition of sea salt and 1.40% *w*/*w* freeze-dried acid whey. ^a–g^ Means within one variable followed by the same letters did not differ significantly (*p* ≥ 0.05).

**Table 7:** Main fractions of fatty acid profile of fermented sausages made from fallow deer and beef meat with different additives (mean ± standard error).

Parameter	Variant	Type of Meat		M	V	M × V
		Beef	Fallow Deer			
SFA (% fatty acid)				***	***	***
	C	56.22 ^b^ ± 0.22	75.23 ^a^ ± 0.30			
	S	55.37 ^b^ ± 0.21	74.67 ^a^ ± 0.29			
	SAW	54.06 ^c^ ± 0.21	74.82 ^a^ ± 0.29			
	SAW2	51.88 ^d^ ± 0.28	74.49 ^a^ ± 0.29			
	SAW4	56.05 ^b^ ± 0.22	74.37 ^a^ ± 0.29			
MUFA (% fatty acid)				***	***	***
	C	38.72 ^d^ ± 0.15	18.23 ^e^ ± 0.07			
	S	39.81 ^c^ ± 0.16	18.62 ^e^ ± 0.07			
	SAW	41.60 ^b^ ± 0.16	18.77 ^e^ ± 0.07			
	SAW2	43.49 ^a^ ± 0.29	18.77 ^e^ ± 0.07			
	SAW4	38.86 ^d^ ± 0.15	18.62 ^e^ ± 0.08			
PUFA (% fatty acid)				***	***	***
	C	5.06 ^e^ ± 0.02	6.48 ^cd^ ± 0.08			
	S	4.82 ^f^ ± 0.03	6.62 ^bc^ ± 0.03			
	SAW	4.33 ^h^ ± 0.02	6.35 ^d^ ± 0.02			
	SAW2	4.61 ^g^ ± 0.04	6.67 ^b^ ± 0.03			
	SAW4	5.08 ^e^ ± 0.03	6.95 ^a^ ± 0.04			

C—sample with addition of sea salt and sodium nitrite; S—sample with addition of sea salt; SAW—sample with addition of sea salt and 0.35% *w*/*w* freeze-dried acid whey; SAW2—sample with addition of sea salt and 0.70% *w*/*w* freeze-dried acid whey; SAW4—sample with addition of sea salt and 1.40% *w*/*w* freeze-dried acid whey. ^a–g^ Means within one variable followed by the same letters did not differ significantly (*p* ≥ 0.05). Significance of fixed effects, M—type of meat, V—variant, M × V—interaction between them, *p*-value: *** (*p* < 0.001), ** (*p* < 0.01), * (*p* < 0.05), n.s. (not significant).

**Table 8:** Fatty acid profile (%) of sausages made from beef and fallow deer.

Fatty Acid	Type of Meat	Variant				
		C	S	SAW	SAW2	SAW4
**C8:0**	Beef	0.02 ^e^ ± 0.00	0.03 ^d^ ± 0.00	0.04 ^d^ ± 0.01	0.05 ^c^ ± 0.00	0.07 ^b^ ± 0.00
	Fallow deer	0.02 ^e^ ± 0.00	0.02 ^e^ ± 0.00	0.03 ^d^ ± 0.00	0.05 ^c^ ± 0.00	0.08 ^a^ ± 0.00
**C10:0**	Beef	0.09 ^de^ ± 0.01	0.09 ^de^ ± 0.00	0.10 ^cd^ ± 0.00	0.12 ^bc^ ± 0.01	0.14 ^a^ ± 0.01
	Fallow deer	0.07 ^fg^ ± 0.01	0.06 ^g^ ± 0.00	0.08 ^ef^ ± 0.00	0.10 ^de^ ± 0.01	0.13 ^ab^ ± 0.00
**C12:0**	Beef	0.10 ^g^ ± 0.00	0.10 ^g^ ± 0.00	0.11 ^g^ ± 0.01	0.13 ^f^ ± 0.01	0.15 ^e^ ± 0.01
	Fallow deer	0.17 ^cd^ ± 0.00	0.16 ^de^ ± 0.00	0.18 ^c^ ± 0.00	0.20 ^b^ ± 0.00	0.23 ^a^ ± 0.00
**C14:0**	Beef	3.71 ^cd^ ± 0.01	3.70 ^cd^ ± 0.06	3.55 ^de^ ± 0.06	3.45 ^e^ ± 0.14	3.80 ^bc^ ± 0.04
	Fallow deer	3.98 ^ab^ ± 0.04	4.01 ^ab^ ± 0.05	4.06 ^a^ ± 0.11	4.04 ^a^ ± 0.10	4.09 ^a^ ± 0.02
**C16:0**	Beef	27.42 ^b^ ± 0.18	27.48 ^b^ ± 0.16	27.48 ^b^ ± 0.16	26.52 ^b^ ± 0.79	27.53 ^b^ ± 0.01
	Fallow deer	29.16 ^a^ ± 0.04	29.04 ^a^ ± 0.05	29.11 ^a^ ± 0.11	28.99 ^a^ ± 0.10	28.78 ^a^ ± 0.02
**C16:1**	Beef	3.73 ^a^ ± 0.76	3.57 ^a^ ± 0.07	4.51 ^a^ ± 0.33	4.12 ^a^ ± 0.76	3.31 ^a^ ± 0.11
	Fallow deer	0.84 ^b^ ± 0.08	0.90 ^b^ ± 0.01	0.94 ^b^ ± 0.01	0.95 ^b^ ± 0.06	0.84 ^b^ ± 0.12
**C18:0**	Beef	24.69 ^c^ ± 0.06	23.78 ^d^ ± 0.25	22.55 ^e^ ± 0.03	21.20 ^f^ ± 0.01	24.16 ^cd^ ± 0.11
	Fallow deer	41.23 ^a^ ± 0.36	40.74 ^ab^ ±0.04	40.70 ^ab^ ±0.18	40.48 ^b^ ± 0.23	40.40 ^b^ ± 0.05
**C18:1**	Beef	34.96 ^d^ ± 0.62	36.27 ^bc^ ± 0.06	37.12 ^b^ ± 0.18	39.63 ^a^ ± 0.36	35.51 ^cd^ ± 0.26
	Fallow deer	17.37 ^e^ ± 0.11	17.70 ^e^ ± 0.02	17.81 ^e^ ± 0.01	17.78 ^e^ ± 0.03	17.81 ^e^ ± 0.04
**C18:2**	Beef	2.93 ^c^ ± 0.05	2.78 ^c^ ± 0.06	2.67 ^c^ ± 0.06	2.69 ^c^ ± 0.08	2.91 ^c^ ± 0.04
	Fallow deer	3.60 ^ab^ ± 0.12	3.69 ^ab^ ± 0.04	3.48 ^b^ ± 0.01	3.69 ^ab^ ± 0.00	3.85 ^a^ ± 0.11
**C18:3 (GLA)**	Beef	0.00	0.00	0.01 ^c^ ± 0.01	0.02 ^bc^ ± 0.00	0.02 ^c^ ± 0.01
	Fallow deer	0.06 ^ab^ ± 0.01	0.08 ^a^ ± 0.02	0.06 ^a^ ± 0.00	0.07 ^a^ ± 0.01	0.07 ^a^ ± 0.01
**C20:0**	Beef	0.17 ^bc^ ± 0.01	0.15 ± 0.01	0.15 ^c^ ± 0.01	0.15 ^bc^ ± 0.00	0.17 ^b^ ± 0.00
	Fallow deer	0.51 ^a^ ± 0.01	0.51 ^a^ ± 0.01	0.52 ^a^ ± 0.01	0.51 ^a^ ± 0.01	0.50 ^a^ ± 0.00
**C18:3 (ALA)**	Beef	1.13 ^a^ ± 0.04	1.02 ^ab^ ± 0.01	0.81 ^cd^ ± 0.00	0.93 ^bc^ ± 0.05	1.13 ^a^ ± 0.04
	Fallow deer	0.75 ^d^ ± 0.06	0.75 ^d^ ± 0.03	0.72 ^d^ ± 0.00	0.76 ^d^ ± 0.00	0.78 ^d^ ± 0.01
**C18:1 (CLA)**	Beef	0.28 ^a^ ± 0.02	0.28 ^a^ ± 0.01	0.25 ^a^ ± 0.08	0.24 ^ab^ ± 0.01	0.28 ^a^ ± 0.02
	Fallow deer	0.13 ^c^ ± 0.00	0.14 ^bc^ ± 0.01	0.19 ^abc^ ± 0.04	0.19 ^abc^ ± 0.03	0.20 ^abc^ ± 0.01
**C22:0**	Beef	0.07 ^b^ ± 0.00	0.06 ^b^ ± 0.01	0.06 ^b^ ± 0.01	0.06 ^b^ ± 0.00	0.07 ^b^ ± 0.00
	Fallow deer	0.17 ^a^ ± 0.01	0.17 ^a^ ± 0.01	0.17 ^a^ ± 0.00	0.17 ^a^ ± 0.00	0.18 ^a^ ± 0.01
**C20:4**	Beef	0.46 ^b^ ± 0.01	0.45 ^b^ ± 0.03	0.39 ^b^ ± 0.01	0.45 ^b^ ± 0.04	0.49 ^b^ ± 0.02
	Fallow deer	1.63 ^a^ ± 0.12	1.74 ^a^ ± 0.01	1.64 ^a^ ± 0.01	1.70 ^a^ ± 0.02	1.76 ^a^ ± 0.04
**C22:1**	Beef	0.01 ^a^ ± 0.00	0.01 ^a^ ± 0.00	0.01 ^a^ ± 0.00	0.01 ^a^ ± 0.00	0.01 ^a^ ± 0.00
	Fallow deer	0.01 ^a^ ± 0.00	0.02 ^a^ ± 0.01	0.02 ^a^ ± 0.00	0.02 ^a^ ± 0.01	0.02 ^a^ ± 0.01
**C20:5 (EPA)**	Beef	0.20 ^ab^ ± 0.01	0.18 ^ab^ ± 0.01	0.15 ^b^ ± 0.00	0.18 ^ab^ ± 0.01	0.21 ^a^ ± 0.01
	Fallow deer	0.15 ^b^ ± 0.04	0.15 ^b^ ± 0.00	0.16 ^ab^ ± 0.00	0.17 ^ab^ ± 0.01	0.17 ^ab^ ± 0.00
**C22:6 (DHA)**	Beef	0.09 ^b^ ± 0.01	0.09 ^b^ ± 0.01	0.08 ^b^ ± 0.01	0.10 ^b^ ± 0.01	0.10 ^b^ ± 0.01
	Fallow deer	0.16 ^a^ ± 0.01	0.16 ^a^ ± 0.00	0.17 ^a^ ± 0.01	0.16 ^a^ ± 0.00	0.16 ^a^ ± 0.00
**UFA/SFA**	Beef	0.78	0.81	0.86	0.94	0.79
	Fallow deer	0.33	0.34	0.34	0.34	0.34
**MUFA/SFA**	Beef	0.69	0.72	0.77	0.85	0.70
	Fallow deer	0.24	0.25	0.25	0.25	0.25
**PUFA/SFA**	Beef	0.09	0.09	0.08	0.09	0.09
	Fallow deer	0.09	0.09	0.09	0.09	0.10
**PUFA 6/3**	Beef	2.42	2.52	2.96	2.62	2.40
	Fallow deer	5.07	5.17	4.96	5.03	5.11

C—sample with addition of sea salt and sodium nitrite; S—sample with addition of sea salt; SAW—sample with addition of sea salt and 0.35% *w*/*w* freeze-dried acid whey; SAW2—sample with addition of sea salt and 0.70% *w*/*w* freeze-dried acid whey; SAW4—sample with addition of sea salt and 1.40% *w*/*w* freeze-dried acid whey. ^a–e^ Means within one variable followed by the same letters did not differ significantly (*p* ≥ 0.05).

**Table 9:** Profile of conjugated linoleic acid (CLA) isomers in fatty acid profile of fermented sausages made from fallow deer and beef meat with different additives (mean ± standard error).

CLA	Variant	Type of Meat		M	V	M × V
		Beef	Fallow Deer			
CLA *cis* 9-*trans*11 (% fatty acid)				***	n.s.	**
	C	0.213 ^ab^ ± 0.003	0.100 ^e^ ± 0.000			
	S	0.237 ^a^ ± 0.003	0.103 ^de^ ± 0.003			
	SAW	0.233 ^a^ ± 0.016	0.133 ^cd^ ± 0.007			
	SAW2	0.187 ^b^ ± 0.003	0.137 ^c^ ± 0.006			
	SAW4	0.220 ^a^ ± 0.005	0.137 ^c^ ± 0.002			
CLA *trans*10-*cis*12 (% fatty acid)				***	**	**
	C	n.d.	0.010 ^b^ ± 0.000			
	S	n.d.	0.013 ^a^ ± 0.001			
	SAW	n.d.	0.013 ^a^ ± 0.001			
	SAW2	n.d.	0.010 ^b^ ± 0.000			
	SAW4	n.d.	0.010 ^b^ ± 0.000			
CLA *cis*9-*cis*11 (% fatty acid)				***	***	***
	C	0.040 ^ab^ ± 0.000	0.010 ^d^ ± 0.000			
	S	0.030 ^c^ ± 0.000	0.010 ^d^ ± 0.000			
	SAW	0.013 ^d^ ± 0.001	0.040 ^ab^ ± 0.000			
	SAW2	0.030 ^c^ ± 0.000	0.030 ^c^ ± 0.000			
	SAW4	0.047 ^a^ ± 0.002	0.037 ^bc^ ± 0.001			
CLA *trans*9-*trans*11 (% fatty acid)				**	***	*
	C	0.020 ^a^ ± 0.004	0.010 ^a^ ± 0.000			
	S	0.010 ^a^ ± 0.000	0.010 ^a^ ± 0.000			
	SAW	0.013 ^ab^ ± 0.001	0.010 ^a^ ± 0.000			
	SAW2	0.020 ^ab^ ± 0.000	0.020 ^ab^ ± 0.000			
	SAW4	0.020 ^ab^ ± 0.000	0.017 ^ab^ ± 0.001			

n.d. (not detected). C—sample with addition of sea salt and sodium nitrite; S—sample with addition of sea salt; SAW—sample with addition of sea salt and 0.35% *w*/*w* freeze-dried acid whey; SAW2—sample with addition of sea salt and 0.70% *w*/*w* freeze-dried acid whey; SAW4—sample with addition of sea salt and 1.40% *w*/*w* freeze-dried acid whey. ^a–e^ Means within one variable followed by the same letters did not differ significantly (*p* ≥ 0.05). Significance of fixed effects, M—type of meat, V—variant, M × V—interaction between them, *p*-value: *** (*p* < 0.001), ** (*p* < 0.01), * (*p* < 0.05), n.s. (not significant).
